# Bis{benzyl *N*′-[(1*H*-indol-3-yl)methyl­ene]dithio­carbazato-κ^2^ *N*′,*S*}copper(II) *N*,*N*-dimethyl­formamide disolvate

**DOI:** 10.1107/S1600536808043808

**Published:** 2009-01-08

**Authors:** Hamid Khaledi, Hapipah Mohd Ali, Seik Weng Ng

**Affiliations:** a Department of Chemistry, University of Malaya, 50603 Kuala Lumpur, Malaysia

## Abstract

In the structure of [Cu(C_17_H_14_N_3_S_2_)_2_]·2C_3_H_7_NO, the Cu atom (site symmetry 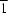) is *N*,*S*-chelated by the two deprotonated Schiff-base anions that define a distorted square-planar geometry. An N—H⋯O hydrogen bond links the mononuclear complex to the DMF solvent mol­ecules.

## Related literature

For the Schiff base ligand, see: Khaledi *et al.* (2008*b* ▶). For the isostructural nickel analog, see: Khaledi *et al.* (2008*a* ▶).
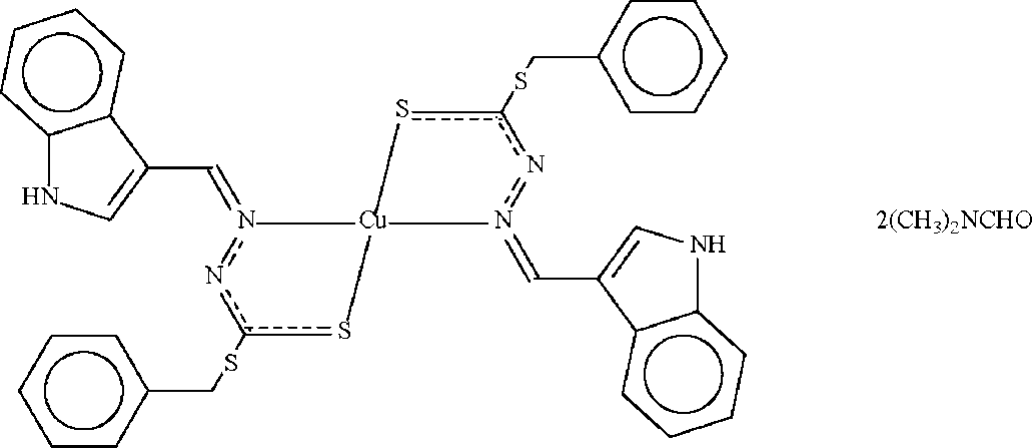


## Experimental

### 

#### Crystal data

[Cu(C_17_H_14_N_3_S_2_)_2_]·2C_3_H_7_NO
*M* *_r_* = 858.60
Monoclinic, 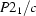

*a* = 10.4461 (2) Å
*b* = 20.0882 (3) Å
*c* = 10.8333 (2) Å
β = 118.366 (1)°
*V* = 2000.34 (6) Å^3^
*Z* = 2
Mo *K*α radiation
μ = 0.80 mm^−1^
*T* = 100 (2) K
0.25 × 0.15 × 0.05 mm

#### Data collection

Bruker SMART APEX diffractometer
Absorption correction: multi-scan (*SADABS*; Sheldrick, 1996 ▶) *T*_min_ = 0.825, *T*_max_ = 0.961
18600 measured reflections
4594 independent reflections
3837 reflections with *I* > 2σ(*I*)
*R*_int_ = 0.035

#### Refinement

*R*[*F* ^2^ > 2σ(*F* ^2^)] = 0.031
*wR*(*F* ^2^) = 0.081
*S* = 1.04
4594 reflections
256 parameters
H atoms treated by a mixture of independent and constrained refinement
Δρ_max_ = 0.36 e Å^−3^
Δρ_min_ = −0.36 e Å^−3^

### 

Data collection: *APEX2* (Bruker, 2007 ▶); cell refinement: *SAINT* (Bruker, 2007 ▶); data reduction: *SAINT*; program(s) used to solve structure: *SHELXS97* (Sheldrick, 2008 ▶); program(s) used to refine structure: *SHELXL97* (Sheldrick, 2008 ▶); molecular graphics: *X-SEED* (Barbour, 2001 ▶); software used to prepare material for publication: *publCIF* (Westrip, 2009 ▶).

## Supplementary Material

Crystal structure: contains datablocks global, I. DOI: 10.1107/S1600536808043808/tk2349sup1.cif

Structure factors: contains datablocks I. DOI: 10.1107/S1600536808043808/tk2349Isup2.hkl

Additional supplementary materials:  crystallographic information; 3D view; checkCIF report

## Figures and Tables

**Table 1:** Hydrogen-bond geometry (Å, °)

*D*—H⋯*A*	*D*—H	H⋯*A*	*D*⋯*A*	*D*—H⋯*A*
N3—H3N⋯O1	0.88 (2)	1.87 (2)	2.742 (2)	175 (2)

**Fig. 1.**
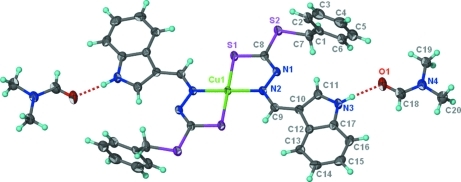
Thermal ellipsoid plot (Barbour, 2001) of Cu(C17H14N3).2DMF at the 70% probability level. Hydrogen atoms are drawn as spheres of arbitrary radius.

## supplementary crystallographic information

### Experimental

Benzyl (1*H*-indol-2-ylmethylene)hydrazinecarbodithioate (Khaledi *et al.*, 2008*b*) (1 mmol, 0.33 g) was dissolved in ethanol (30 ml). To the clear solution was added an ethanol solution (10 ml) containing 1 mmol (0.09 g) of copper chloride dihydrate. The mixture was heated for an hour. The product that separated was recrystallized from DMF.

### Refinement

The C-bound H atoms were placed at calculated positions (C–H 0.95–0.99 Å) and were treated as riding on their parent C atoms, with *U*(H) set to 1.2–1.5 times *U*_eq_(*C*). The amino H-atom was located in a difference Fourier map, and was refined with a distance restraint of N–H 0.88±0.01 Å; its temperature factor was freely refined.

### Crystal data

**Table:**

[Cu(C_17_H_14_N_3_S_2_)_2_]·2C_3_H_7_NO	*Z* = 2
*M**_r_* = 858.60	*F*(000) = 894
Monoclinic, *P*2_1_/*c*	*D*_x_ = 1.425 Mg m^−^^3^
Hall symbol: -P 2ybc	Mo *K*α radiation, λ = 0.71073 Å
*a* = 10.4461 (2) Å	µ = 0.80 mm^−^^1^
*b* = 20.0882 (3) Å	*T* = 100 K
*c* = 10.8333 (2) Å	Irregular block, brown
β = 118.366 (1)°	0.25 × 0.15 × 0.05 mm
*V* = 2000.34 (6) Å^3^	

### Data collection

**Table:**

Bruker SMART APEX diffractometer	4594 independent reflections
Radiation source: fine-focus sealed tube	3837 reflections with *I* > 2σ(*I*)
graphite	*R*_int_ = 0.035
ω scans	θ_max_ = 27.5°, θ_min_ = 2.0°
Absorption correction: multi-scan (*SADABS*; Sheldrick, 1996)	*h* = −13→13
*T*_min_ = 0.825, *T*_max_ = 0.961	*k* = −25→26
18600 measured reflections	*l* = −14→14

### Refinement

**Table:**

Refinement on *F*^2^	Primary atom site location: structure-invariant direct methods
Least-squares matrix: full	Secondary atom site location: difference Fourier map
*R*[*F*^2^ > 2σ(*F*^2^)] = 0.031	Hydrogen site location: inferred from neighbouring sites
*wR*(*F*^2^) = 0.081	H atoms treated by a mixture of independent and constrained refinement
*S* = 1.04	*w* = 1/[σ^2^(*F*_o_^2^) + (0.0382*P*)^2^ + 0.7321*P*] where *P* = (*F*_o_^2^ + 2*F*_c_^2^)/3
4594 reflections	(Δ/σ)_max_ = 0.001
256 parameters	Δρ_max_ = 0.36 e Å^−^^3^
0 restraints	Δρ_min_ = −0.36 e Å^−^^3^

### Fractional atomic coordinates and isotropic or equivalent isotropic displacement parameters (Å^2^)

**Table:**

	*x*	*y*	*z*	*U*_iso_*/*U*_eq_	
Cu1	0.5000	0.5000	0.5000	0.01678 (8)	
O1	0.8350 (2)	0.56467 (8)	−0.10421 (17)	0.0526 (5)	
S1	0.51561 (5)	0.61250 (2)	0.51610 (5)	0.02414 (11)	
S2	0.61468 (5)	0.70543 (2)	0.37409 (5)	0.02345 (11)	
N1	0.60380 (15)	0.57545 (7)	0.32793 (14)	0.0191 (3)	
N2	0.56823 (15)	0.51211 (7)	0.35752 (15)	0.0193 (3)	
N3	0.72111 (17)	0.49444 (7)	0.03797 (16)	0.0234 (3)	
H3N	0.756 (3)	0.5192 (11)	−0.006 (2)	0.040 (6)*	
N4	0.91692 (17)	0.57480 (8)	−0.26314 (16)	0.0267 (3)	
C1	0.81749 (19)	0.68443 (8)	0.27140 (19)	0.0223 (4)	
C2	0.9316 (2)	0.68264 (9)	0.4074 (2)	0.0282 (4)	
H2	0.9149	0.6937	0.4838	0.034*	
C3	1.0699 (2)	0.66472 (10)	0.4321 (2)	0.0338 (4)	
H3	1.1469	0.6628	0.5258	0.041*	
C4	1.0974 (2)	0.64964 (10)	0.3226 (2)	0.0340 (5)	
H4	1.1926	0.6376	0.3406	0.041*	
C5	0.9852 (2)	0.65223 (10)	0.1870 (2)	0.0315 (4)	
H5	1.0030	0.6423	0.1108	0.038*	
C6	0.8463 (2)	0.66931 (9)	0.1618 (2)	0.0261 (4)	
H6	0.7695	0.6707	0.0680	0.031*	
C7	0.6635 (2)	0.70369 (9)	0.23471 (19)	0.0245 (4)	
H7A	0.5968	0.6724	0.1620	0.029*	
H7B	0.6446	0.7485	0.1915	0.029*	
C8	0.58034 (17)	0.62208 (8)	0.39595 (17)	0.0188 (3)	
C9	0.58804 (18)	0.46384 (8)	0.28764 (18)	0.0204 (3)	
H9	0.5641	0.4209	0.3071	0.024*	
C10	0.63940 (18)	0.46470 (8)	0.18750 (17)	0.0201 (3)	
C11	0.68033 (19)	0.51762 (9)	0.13067 (19)	0.0229 (4)	
H11	0.6796	0.5632	0.1538	0.027*	
C12	0.65845 (18)	0.40510 (8)	0.12243 (17)	0.0196 (3)	
C13	0.63965 (19)	0.33709 (9)	0.13551 (18)	0.0234 (4)	
H13	0.6062	0.3216	0.1980	0.028*	
C14	0.6707 (2)	0.29284 (9)	0.0556 (2)	0.0276 (4)	
H14	0.6595	0.2464	0.0645	0.033*	
C15	0.7185 (2)	0.31520 (9)	−0.0383 (2)	0.0285 (4)	
H15	0.7380	0.2837	−0.0926	0.034*	
C16	0.73753 (19)	0.38186 (9)	−0.05314 (18)	0.0250 (4)	
H16	0.7690	0.3972	−0.1173	0.030*	
C17	0.70879 (18)	0.42596 (9)	0.02967 (18)	0.0211 (3)	
C18	0.8710 (3)	0.54041 (11)	−0.1878 (2)	0.0395 (5)	
H18	0.8652	0.4934	−0.1989	0.047*	
C19	0.9269 (2)	0.64686 (9)	−0.2527 (2)	0.0294 (4)	
H19A	0.9223	0.6613	−0.1685	0.044*	
H19B	0.8460	0.6666	−0.3358	0.044*	
H19C	1.0193	0.6613	−0.2467	0.044*	
C20	0.9516 (2)	0.54244 (10)	−0.3636 (2)	0.0314 (4)	
H20A	0.9441	0.4941	−0.3573	0.047*	
H20B	1.0510	0.5541	−0.3427	0.047*	
H20C	0.8832	0.5573	−0.4587	0.047*	

### Atomic displacement parameters (Å^2^)

**Table:**

	*U*^11^	*U*^22^	*U*^33^	*U*^12^	*U*^13^	*U*^23^
Cu1	0.01726 (15)	0.01781 (15)	0.01939 (15)	0.00000 (11)	0.01205 (12)	0.00001 (11)
O1	0.0872 (13)	0.0476 (10)	0.0468 (9)	−0.0192 (9)	0.0511 (10)	−0.0044 (7)
S1	0.0345 (3)	0.0189 (2)	0.0316 (2)	−0.00003 (17)	0.0260 (2)	−0.00067 (17)
S2	0.0308 (2)	0.0175 (2)	0.0305 (2)	0.00151 (17)	0.0214 (2)	0.00143 (17)
N1	0.0217 (7)	0.0178 (7)	0.0208 (7)	−0.0002 (5)	0.0126 (6)	0.0020 (6)
N2	0.0222 (7)	0.0179 (7)	0.0214 (7)	−0.0007 (5)	0.0134 (6)	0.0007 (5)
N3	0.0284 (8)	0.0236 (8)	0.0266 (8)	0.0016 (6)	0.0198 (7)	0.0027 (6)
N4	0.0282 (8)	0.0283 (8)	0.0251 (8)	−0.0006 (6)	0.0138 (7)	0.0045 (6)
C1	0.0268 (9)	0.0148 (8)	0.0302 (9)	−0.0002 (7)	0.0175 (8)	0.0050 (7)
C2	0.0309 (10)	0.0290 (10)	0.0281 (9)	−0.0024 (8)	0.0168 (8)	0.0022 (8)
C3	0.0247 (10)	0.0349 (11)	0.0346 (11)	−0.0042 (8)	0.0082 (9)	0.0018 (9)
C4	0.0256 (10)	0.0290 (10)	0.0506 (13)	−0.0022 (8)	0.0207 (10)	−0.0036 (9)
C5	0.0329 (11)	0.0303 (10)	0.0405 (11)	−0.0016 (8)	0.0249 (10)	−0.0031 (8)
C6	0.0285 (9)	0.0252 (9)	0.0290 (9)	−0.0006 (7)	0.0172 (8)	0.0027 (7)
C7	0.0291 (9)	0.0236 (9)	0.0274 (9)	0.0054 (7)	0.0187 (8)	0.0081 (7)
C8	0.0158 (8)	0.0215 (8)	0.0196 (8)	0.0016 (6)	0.0090 (7)	0.0025 (6)
C9	0.0223 (9)	0.0182 (8)	0.0240 (9)	−0.0011 (6)	0.0137 (7)	−0.0003 (7)
C10	0.0214 (8)	0.0197 (8)	0.0212 (8)	−0.0004 (6)	0.0118 (7)	−0.0010 (7)
C11	0.0262 (9)	0.0219 (8)	0.0272 (9)	0.0022 (7)	0.0182 (8)	0.0003 (7)
C12	0.0178 (8)	0.0226 (8)	0.0197 (8)	0.0000 (6)	0.0100 (7)	−0.0026 (7)
C13	0.0223 (9)	0.0239 (9)	0.0259 (9)	−0.0034 (7)	0.0129 (8)	−0.0027 (7)
C14	0.0238 (9)	0.0227 (9)	0.0355 (10)	−0.0022 (7)	0.0135 (8)	−0.0060 (8)
C15	0.0254 (9)	0.0303 (10)	0.0310 (10)	0.0022 (8)	0.0143 (8)	−0.0098 (8)
C16	0.0223 (9)	0.0340 (10)	0.0220 (9)	0.0023 (7)	0.0131 (8)	−0.0034 (7)
C17	0.0192 (8)	0.0237 (9)	0.0210 (8)	0.0025 (7)	0.0101 (7)	−0.0001 (7)
C18	0.0543 (14)	0.0337 (11)	0.0362 (11)	−0.0107 (10)	0.0262 (11)	0.0016 (9)
C19	0.0338 (11)	0.0283 (10)	0.0271 (10)	−0.0031 (8)	0.0154 (9)	0.0030 (8)
C20	0.0308 (10)	0.0354 (11)	0.0312 (10)	0.0037 (8)	0.0173 (9)	0.0028 (8)

### Geometric parameters (Å, °)

**Table:**

Cu1—N2	1.9987 (14)	C5—H5	0.9500
Cu1—N2^i^	1.9987 (14)	C6—H6	0.9500
Cu1—S1	2.2666 (4)	C7—H7A	0.9900
Cu1—S1^i^	2.2666 (4)	C7—H7B	0.9900
O1—C18	1.234 (3)	C9—C10	1.421 (2)
S1—C8	1.7392 (17)	C9—H9	0.9500
S2—C8	1.7519 (17)	C10—C11	1.394 (2)
S2—C7	1.8092 (17)	C10—C12	1.450 (2)
N1—C8	1.285 (2)	C11—H11	0.9500
N1—N2	1.4048 (18)	C12—C17	1.401 (2)
N2—C9	1.306 (2)	C12—C13	1.397 (2)
N3—C11	1.347 (2)	C13—C14	1.382 (2)
N3—C17	1.381 (2)	C13—H13	0.9500
N3—H3N	0.88 (2)	C14—C15	1.403 (3)
N4—C18	1.321 (2)	C14—H14	0.9500
N4—C19	1.452 (2)	C15—C16	1.374 (3)
N4—C20	1.454 (2)	C15—H15	0.9500
C1—C6	1.390 (2)	C16—C17	1.391 (2)
C1—C2	1.387 (3)	C16—H16	0.9500
C1—C7	1.512 (2)	C18—H18	0.9500
C2—C3	1.386 (3)	C19—H19A	0.9800
C2—H2	0.9500	C19—H19B	0.9800
C3—C4	1.380 (3)	C19—H19C	0.9800
C3—H3	0.9500	C20—H20A	0.9800
C4—C5	1.378 (3)	C20—H20B	0.9800
C4—H4	0.9500	C20—H20C	0.9800
C5—C6	1.386 (3)		
			
N2—Cu1—N2^i^	180.000 (1)	S1—C8—S2	112.60 (9)
N2—Cu1—S1	84.21 (4)	N2—C9—C10	130.93 (16)
N2^i^—Cu1—S1	95.79 (4)	N2—C9—H9	114.5
N2—Cu1—S1^i^	95.79 (4)	C10—C9—H9	114.5
N2^i^—Cu1—S1^i^	84.21 (4)	C11—C10—C9	130.77 (16)
S1—Cu1—S1^i^	180.0	C11—C10—C12	105.86 (15)
C8—S1—Cu1	95.28 (6)	C9—C10—C12	123.36 (15)
C8—S2—C7	104.55 (8)	N3—C11—C10	109.74 (15)
C8—N1—N2	112.72 (13)	N3—C11—H11	125.1
C9—N2—N1	114.11 (14)	C10—C11—H11	125.1
C9—N2—Cu1	124.77 (12)	C17—C12—C13	118.93 (15)
N1—N2—Cu1	121.10 (10)	C17—C12—C10	106.66 (15)
C11—N3—C17	109.91 (15)	C13—C12—C10	134.40 (16)
C11—N3—H3N	124.5 (15)	C14—C13—C12	118.64 (16)
C17—N3—H3N	125.5 (15)	C14—C13—H13	120.7
C18—N4—C19	120.47 (17)	C12—C13—H13	120.7
C18—N4—C20	121.44 (17)	C13—C14—C15	121.20 (17)
C19—N4—C20	118.02 (15)	C13—C14—H14	119.4
C6—C1—C2	118.43 (17)	C15—C14—H14	119.4
C6—C1—C7	117.81 (16)	C16—C15—C14	121.23 (17)
C2—C1—C7	123.75 (16)	C16—C15—H15	119.4
C3—C2—C1	120.16 (18)	C14—C15—H15	119.4
C3—C2—H2	119.9	C15—C16—C17	117.19 (17)
C1—C2—H2	119.9	C15—C16—H16	121.4
C4—C3—C2	120.95 (18)	C17—C16—H16	121.4
C4—C3—H3	119.5	N3—C17—C16	129.38 (16)
C2—C3—H3	119.5	N3—C17—C12	107.84 (14)
C3—C4—C5	119.34 (18)	C16—C17—C12	122.79 (16)
C3—C4—H4	120.3	O1—C18—N4	125.0 (2)
C5—C4—H4	120.3	O1—C18—H18	117.5
C4—C5—C6	119.94 (18)	N4—C18—H18	117.5
C4—C5—H5	120.0	N4—C19—H19A	109.5
C6—C5—H5	120.0	N4—C19—H19B	109.5
C5—C6—C1	121.17 (18)	H19A—C19—H19B	109.5
C5—C6—H6	119.4	N4—C19—H19C	109.5
C1—C6—H6	119.4	H19A—C19—H19C	109.5
C1—C7—S2	118.16 (12)	H19B—C19—H19C	109.5
C1—C7—H7A	107.8	N4—C20—H20A	109.5
S2—C7—H7A	107.8	N4—C20—H20B	109.5
C1—C7—H7B	107.8	H20A—C20—H20B	109.5
S2—C7—H7B	107.8	N4—C20—H20C	109.5
H7A—C7—H7B	107.1	H20A—C20—H20C	109.5
N1—C8—S1	126.61 (13)	H20B—C20—H20C	109.5
N1—C8—S2	120.80 (13)		
			
N2—Cu1—S1—C8	−1.75 (7)	Cu1—N2—C9—C10	−177.95 (14)
N2^i^—Cu1—S1—C8	178.25 (7)	N2—C9—C10—C11	−2.0 (3)
C8—N1—N2—C9	178.63 (15)	N2—C9—C10—C12	178.22 (17)
C8—N1—N2—Cu1	−3.25 (19)	C17—N3—C11—C10	0.1 (2)
S1—Cu1—N2—C9	−179.02 (14)	C9—C10—C11—N3	−179.57 (17)
S1^i^—Cu1—N2—C9	0.98 (14)	C12—C10—C11—N3	0.2 (2)
S1—Cu1—N2—N1	3.07 (11)	C11—C10—C12—C17	−0.39 (19)
S1^i^—Cu1—N2—N1	−176.93 (11)	C9—C10—C12—C17	179.40 (15)
C6—C1—C2—C3	−1.4 (3)	C11—C10—C12—C13	178.57 (19)
C7—C1—C2—C3	−179.70 (17)	C9—C10—C12—C13	−1.6 (3)
C1—C2—C3—C4	1.2 (3)	C17—C12—C13—C14	−0.4 (2)
C2—C3—C4—C5	−0.3 (3)	C10—C12—C13—C14	−179.23 (18)
C3—C4—C5—C6	−0.5 (3)	C12—C13—C14—C15	−0.8 (3)
C4—C5—C6—C1	0.4 (3)	C13—C14—C15—C16	0.7 (3)
C2—C1—C6—C5	0.6 (3)	C14—C15—C16—C17	0.6 (3)
C7—C1—C6—C5	179.01 (16)	C11—N3—C17—C16	179.22 (18)
C6—C1—C7—S2	166.51 (13)	C11—N3—C17—C12	−0.3 (2)
C2—C1—C7—S2	−15.1 (2)	C15—C16—C17—N3	178.73 (17)
C8—S2—C7—C1	−80.69 (15)	C15—C16—C17—C12	−1.8 (3)
N2—N1—C8—S1	1.3 (2)	C13—C12—C17—N3	−178.72 (15)
N2—N1—C8—S2	−179.21 (11)	C10—C12—C17—N3	0.43 (18)
Cu1—S1—C8—N1	0.81 (16)	C13—C12—C17—C16	1.7 (3)
Cu1—S1—C8—S2	−178.73 (8)	C10—C12—C17—C16	−179.15 (16)
C7—S2—C8—N1	5.62 (16)	C19—N4—C18—O1	−0.7 (3)
C7—S2—C8—S1	−174.81 (9)	C20—N4—C18—O1	−177.5 (2)
N1—N2—C9—C10	0.1 (3)		

Symmetry codes: (i) −*x*+1, −*y*+1, −*z*+1.

### Hydrogen-bond geometry (Å, °)

**Table:**

*D*—H···*A*	*D*—H	H···*A*	*D*···*A*	*D*—H···*A*
N3—H3N···O1	0.88 (2)	1.87 (2)	2.742 (2)	175 (2)
